# The safety and feasibility of intraoperative near‐infrared fluorescence imaging with indocyanine green in thoracoscopic sympathectomy for primary palmar hyperhidrosis

**DOI:** 10.1111/1759-7714.13345

**Published:** 2020-02-15

**Authors:** Guotian Pei, Yanguo Liu, Qiang Liu, Xianjun Min, Yingshun Yang, Shuai Wang, Jun Liu, Jun Wang, Yuqing Huang

**Affiliations:** ^1^ Department of Thoracic Surgery Beijing Haidian Hospital (Haidian Section of Peking University Third Hospital) Beijing China; ^2^ Department of Thoracic Surgery Peking University People's Hospital Beijing China

**Keywords:** Fluorescence imaging, hyperhidrosis, indocyanine green, sympathectomy

## Abstract

**Background:**

We investigated the safety and feasibility of intraoperative near‐infrared (NIR) imaging using indocyanine green (ICG) during sympathectomy in the management of primary palmar hyperhidrosis (PPH).

**Methods:**

We performed a retrospective review of 142 patients (ICG group) who underwent endoscopic thoracic sympathectomy (ETS) between February 2018 and April 2019. All patients received a 5 mg/kg infusion of ICG 24 hours preoperatively. The vital signs before and after ICG injection and adverse reactions were recorded. Meanwhile, 498 patients (Non‐ICG group) who underwent ETS by normal thoracoscopy during August 2017 to April 2019 were also reviewed to compare the abnormal white blood cell (WBC) counts, alanine transaminase (ALT), aspartate transaminase (AST), blood urea nitrogen (BUN), and creatinine (Cr) levels before and after operation between two groups.

**Results:**

For ICG group, the vital signs including body temperature, heart rate and blood pressure before and after ICG injection were stable. There was no significant difference in the abnormal WBC counts, ALT, AST, BUN, and Cr levels before and after operation between two groups. Only one patient had mild adverse reaction (0.7%) after ICG injection. The visibility rate of all sympathetic ganglions was 96.7% (1369/1415). The visibility rate from T1 to T5 was 98.23% (278/283), 98.23% (278/283), 97.17% (275/283), 95.76% (271/283), and 94.35% (267/283), respectively. There was no significant difference in the visibility rate with regard to age, gender, height, weight, body mass index, and PPH grade.

**Conclusions:**

NIR fluorescence imaging with ICG for identifying sympathetic ganglions is relatively safe and feasible.

**Key points:**

• **Significant findings of the study.**

NIR fluorescence imaging with ICG for identifying sympathetic ganglions is relatively safe and feasible.

• **What this study adds**.

This technology may take the place of the rib‐oriented method as standard practice for the precise localization of sympathetic ganglions, and may improve the effect of sympathectomies.

## Introduction

Primary palmar hyperhidrosis (PPH) is a disorder characterized by excessive sweating which can cause significant professional and social handicaps. Endoscopic thoracic sympathectomy (ETS) has been determined as the gold standard of surgical treatment for PPH.^1^ However, the results and side effects of this procedure vary between patients. One cause of this phenomenon is the anatomical variation of the sympathetic ganglion, which is commonly invisible on normal thoracoscopy. Intraoperative near‐infrared (NIR) fluorescence imaging with indocyanine green (ICG) has been used for many thoracic surgeries. ICG is considered a safe dye with a low incidence of adverse reactions.^2^, ^3^, ^4^ The sympathetic ganglion has been successfully visualized using infrared thoracoscopy with an injection of ICG.^5^ Patients received a 5 mg/kg infusion of ICG 24 hours prior to surgery which is a 2.5 to 10 times higher dosage than recommended for cardiac output and liver function monitoring approved by the US Food and Drug Administration (FDA).^6^ However, ICG does not have a thoracic FDA indication. Thus, the safety and feasibility of ICG application is still controversially discussed. The primary objective of this study was to demonstrate the safety and feasibility of intraoperative NIR imaging using ICG during sympathectomy in the management of primary palmar hyperhidrosis. This study has previously been presented as an oral presentation at the 13th ISSS (International Society of Sympathetic Surgery) World Symposium (https://www.isssitaly2019.org).

## Methods

### Patients

This retrospective study included 142 consecutive eligible patients with a diagnosis of PPH treated by thoracoscopic sympathectomy using the FloNavi Endoscopic Fluorescence Imaging System in the Department of Thoracic Surgery at Peking University Peoples Hospital and Beijing Haidian Hospital (Haidian Section of Peking University Third Hospital) from February 2018 to April 2019. Inclusion criteria were as follow: (i) Patients were aged between 18–75 years old; (ii) palmar sweating was the major complaint with or without axillary and plantar sweating; (iii) patients without an allergy to iodine or ICG. All patients provided their voluntary informed consent for the use of preoperative intravenous ICG injection. These patients were classified as the ICG group. Vital signs, adverse reactions, routine haematological examinations, and hepatic and renal functions tests were tested before and after ICG injection in order to assess the safety of ICG. Routine haematological examinations, and hepatic and renal function tests of all patients were tested on admission and the first day postoperatively. The vital signs of ICG group patients were tested on admission and after ICG injection. The major observation items before and after ICG injection included body temperature, heart rate, blood pressure, abnormal white blood cell (WBC, 3.5–9.5 × 109/L) counts, alanine transaminase (ALT, 9–50 U/L), aspartate transaminase (AST, 15–40 U/L), blood urea nitrogen (BUN, 44–123 IU/L), and creatinine (Cr, 2.86–8.2 mmol/L) levels. The adverse reactions were categorized into three levels as previously described^7^: mild, moderate, and severe, which were dependent on the duration, need for medical intervention, and final outcomes after the allergic reaction had occurred.

To better evaluate the safety of intraoperative NIR fluorescence imaging with ICG in thoracoscopic sympathectomy for PPH, we reviewed the files of 498 patients who had undergone ETS by normal thoracoscopy from August 2017 to April 2019. These patients were classified as the Non‐ICG group. The abnormal WBC counts, ALT, AST, BUN and Cr levels before and after operation were compared between the two groups.

The preoperative workup was standardized and included clinical examination, laboratory investigations, electrocardiogram examination and chest radiograph. All patients were asked to fill in the self‐rating depression scale (SDS), self‐rating anxiety scale (SAS), and personality diagnostic questionnaire (PDQ‐4) in order to determine their psychological and personality characteristics. The medical records of each patient were reviewed and demographic and clinical data recorded including age, gender, height, weight, BMI, grade of PPH, and adverse reactions. The study was approved by the Medical Ethics Committees of the participating institutes.

### Fluorescence endoscopic imaging system

**Figure 1:**
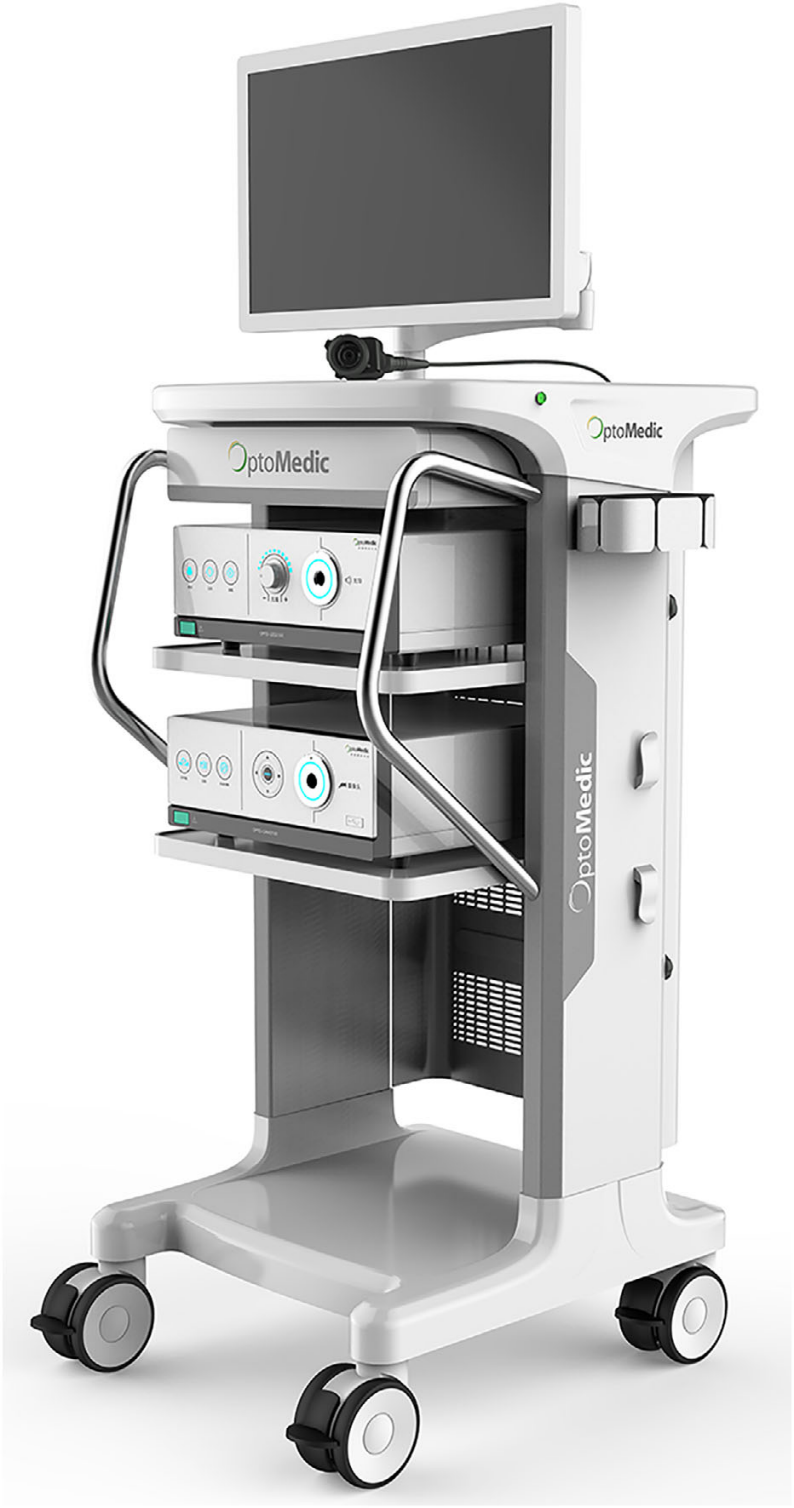
The FloNavi endoscopic fluorescence imaging system.

**Figure 2:**
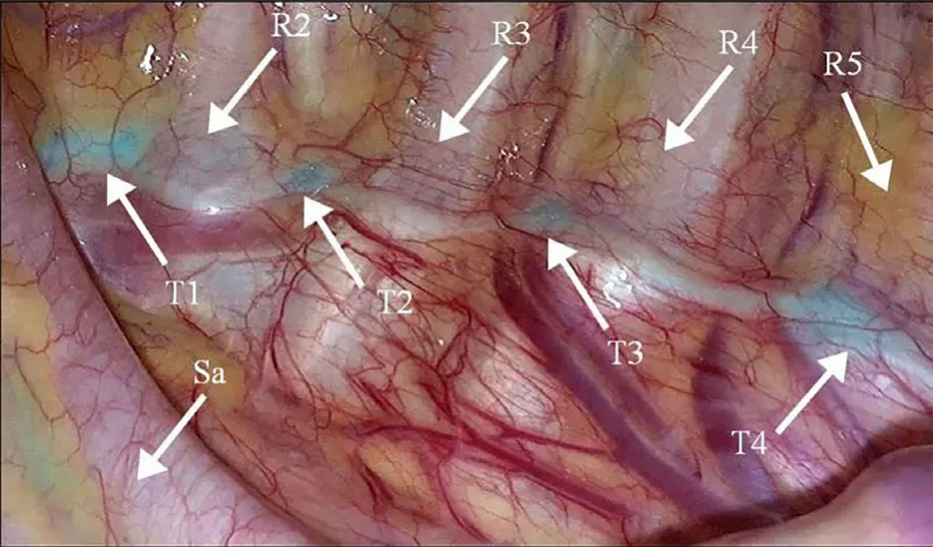
A representative image of the sympathetic ganglions under NIR fluorescent thoracoscopy.

Intraoperative imaging procedures were performed using the FloNavi endoscopic fluorescence imaging system (Optomedic Technique Inc., Guangdong, China) (Fig 1), which provided near‐infrared laser to excite the ICG agent and capture the excited fluorescent signal. Tissues in the field of view were simultaneously irradiated by visible light and the 805 nm excitation laser. The NIR fluorescence and the visible light reflected by the tissues were captured by the complementary metal‐oxide semiconductor (CMOS) component, while the exciting light was filtered out by a long‐pass filter. In order to visualize the tissue with fluorescence, the NIR signal was extracted for original exhibition and fused with visible signal for more visual information. A representative image of the sympathetic ganglions under NIR fluorescent thoracoscopy is shown in Fig 2.

### Surgical procedure

For the Non‐ICG group, the surgical technique of ETS was performed as previously described.^8^ For the ICG group, all patients received 5 mg/kg of intravenous ICG infusion (Tian Yi Pharmaceutical Co., Ltd., Liaoning Province of China, 25 mg) 24 hours preoperatively. Before ICG injection, methylprednisolone (40 mg) was used as a premedication for all patients. All procedures were performed by uniportal thoracoscopy under general anesthesia using a laryngeal mask airway. Patients were placed in a semi‐Fowler's position, and a 1.5 cm incision was made in the fourth intercostal space on the middle axillary line of both sides. During the surgical procedure, the sympathetic chain was first identified with a white light and then observed under NIR light. The sympathetic ganglions were recognized from top to bottom, and the sympathetic chain was then amputated by electrocautery regardless of whether the sympathetic ganglions were visible or not. That is to say, the imaging results of the sympathetic ganglions during operation did not guide the choice of the surgical procedures in this study. The transection range was laterally extended approximately 2 cm along the surface of the corresponding rib in order to amputate the potential bypass nerve fibers. A chest radiograph was requested on the day of surgery to ensure complete lung expansion. All operations were performed by the same surgeon (Yanguo Liu). A grading scale was used to assess the intraoperative visibility rate of the sympathetic ganglions (0, agent not observed; 1, agent observed with difficulty; 2, agent easily observed), developed by Price *et al*.^9^ The intraoperative visibility rate of the sympathetic ganglions was graded by another two surgeons.

### Statistical analysis

The data are presented as means and standard deviations for continuous variables and as frequency and percentage for categorical variables. Student *t*‐tests were used to compare continuous variables and the χ^2^ or Fisher exact tests were used for categorical variables. The kappa (*κ*) statistic was used to evaluate the level of agreement between two surgeons for the visibility grade of sympathetic ganglions. The strength of agreement for the *κ* value was interpreted as follows: *κ* < 0.01 = poor, 0.01–0.20 = slight, 0.21–0.40 = fair, 0.41–0.60 = moderate, 0.61–0.80 = substantial, and 0.81–0.99 = almost perfect.^10^ Univariate and multivariate logistic regression analyses were used to detect relevant factors for invisibility of the sympathetic ganglions. All statistical analyses were performed using SPSS 13.0 (SPSS Inc., Chicago, IL) software and a *P‐*value less than 0.05 was considered to be statistically significant.

## Results

For the ICG group, a total of 142 consecutive patients (74 males and 68 females) were enrolled into this study. All patients underwent bilateral sympathectomy apart from one patient with severe pleural adhesions. The patients’ characteristics are summarized in Table 1. ICG injection was possible in all patients, and the vital signs including body temperature, heart rate and blood pressure before and after ICG injection were stable. There were no statistically significant changes before and after ICG injection in terms of body temperature, heart rate and blood pressure (Table 2). The abnormal WBC counts, ALT, AST, BUN and Cr levels before and after operation are summarized in Table 3. There were no statistically significant differences in abnormal rates between the ICG group and the Non‐ICG group. Only one patient had a mild adverse reaction of subcutaneous extravasation after the ICG injection which resulted in a slightly stinging‐like pain and a small area of skin which changed in color, but there was no tissue necrosis. Telephone follow‐up was performed and patients reported that they had recovered and were back to normal two weeks after the operation. The incidence of mild adverse reaction was 0.70%. There were no moderate or severe adverse reactions after ICG administration.

**Table 1:** Characteristics of patients

	ICG group (*n* = 142)	Non‐ICG group (*n* = 498)	*P*‐value
Age, years	26.40 ± 5.97	25.34 ± 7.05	0.104
Gender (F:M), *n* (%)	68 (47.9): 74 (52.1)	243 (48.8): 255 (51.2)	0.486
Height, m	1.68 ± 0.08	1.68 ± 0.08	0.803
Weight, kg	61.58 ± 10.88	60.77 ± 10.87	0.437
BMI, kg/m^2^	21.68 ± 3.11	21.48 ± 3.62	0.535
Grade of PPH, *n* (%)			0.966
Mild	9 (6.34)	30(6.02)	
Moderate	52 (36.62)	178(35.74)	
Severe	81 (57.04)	290 (58.23)	

BMI, body mass index; ICG, indocyanine green; PPH, primary palmar hyperhidrosis.

**Table 2:** The vital signs before and after ICG injection

	Before ICG	After ICG	*P*‐value
Body temperature	36.44 ± 0.31	36.37 ± 0.26	0.131
Heart rate	78.19 ± 10.51	79.70 ± 7.94	0.081
Blood pressure			
Systolic pressure	126.29 ± 11.27	126.10 ± 13.48	0.863
Diastolic pressure	78.29 ± 8.71	78.85 ± 7.10	0.424

ICG, indocyanine green

**Table 3:** The abnormal WBC counts, ALT, AST, BUN and Cr levels before and after operation between two groups

	Non‐ICG group		ICG group		*P*‐value
	*n*	Abnormal rate	*n*	Abnormal rate	
WBC					
Preoperative	34/498	6.83	8/142	5.63	0.61
Postoperative	342/498	68.67	106/142	74.65	0.18
ALT					
Preoperative	11/498	2.21	4/142	2.82	0.67
Postoperative	6/498	1.20	0/142	0	0.35
AST					
Preoperative	5/498	1.00	1/142	0.70	0.74
Postoperative	0/498	0	0/142	0	‐
BUN					
Preoperative	0/498	0	0/142	0	‐
Postoperative	0/498	0	0/142	0	‐
Cr					
Preoperative	1/498	0.20	0/142	0	1.00
Postoperative	4/498	0.80	2/142	1.41	0.62

ALT, alanine transaminase; AST, aspartate transaminase; BUN, blood urea nitrogen; Cr, creatinine; ICG, indocyanine green; WBC, white blood cells.

A total of 1369 sympathetic ganglions were identified successfully during surgery in the ICG group. The visibility rate of all sympathetic ganglions was 96.7% (1369/1415). The visibility rate from T1 to T5 was 98.23% (278/283), 98.23% (278/283), 97.17% (275/283), 95.76% (271/283), and 94.35% (267/283), respectively. There was no significant difference in visibility rate with regard to age, gender, height, weight, BMI, and PPH grade (Table 4). The visibility grade of sympathetic ganglions from T1 to T5 between two surgeons was in a good agreement by the kappa value (*κ*) of 1.000, 1.000, 0.951, 0.905, and 0.876, respectively (Table 5).

**Table 4:** Factors influencing the visibility rate of sympathetic ganglions estimated by univariate and multivariate linear regression

	Univariate regression		Multivariate regression	
	Effect (95% CI)	*P*‐value	Effect (95% CI)	*P*‐value
Age	1.027 (0.942–1.120)	0.545	1.014 (0.925–1.111)	0.769
Gender	1.024 (0.389–2.695)	0.961	0.868 (0.175–4.303)	0.862
Height	0.990 (0.932–1.051)	0.742	1.314 (0.844–2.047)	0.227
Weight	0.989 (0.947–1.033)	0.628	0.680 (0.374–1.236)	0.205
BMI	0.984 (0.847–1.144)	0.838	2.893 (0.499–16.760)	0.236
Grade of the PPH				
Mild	Reference		Reference	
Moderate	0.958 (0.101–9.058)	0.970	1.201 (0.121–11.883)	0.876
Severe	0.719 (0.082–6.278)	0.765	0.733 (0.081–6.635)	0.783

BMI, body mass index; PPH, primary palmar hyperhidrosis.

**Table 5:** Agreement in visibility grade of the sympathetic ganglion between two surgeons

		Surgeon (X.M)																			
		T1				T2				T3				T4				T5			
Surgeon (Y.Y)	Grade	0	1	2		0	1	2		0	1	2		0	1	2		0	1	2	
	0	5	0	0	5	5	0	0	5	8	0	0	8	12	0	0	12	16	1	0	17
	1	0	0	0	0	0	0	0	0	0	2	1	3	0	3	1	4	1	2	2	5
	2	0	0	278	278	0	0	278	278	0	0	272	272	0	2	265	267	0	1	260	261
		5	0	278	278	5	0	278	278	8	2	273	283	12	5	266	283	17	4	263	283
		κ 1.000 95% CI 0.000–16.823.				κ 1.000 95% CI 0.000–16.823.				κ 0.951 95% CI 0.049–18.866.				κ 0.905 95% CI 0.054– 18.312				κ 0.876 95% CI 0.052– 17.152			

## Discussion

ETS has been proven to be a safe and effective treatment for PPH. However, the results and side effects of this procedure vary between patients. One of the most important reasons for this phenomenon is anatomical variation of the sympathetic nerve such as the sympathetic trunk pathway, the position of the sympathetic ganglion, and accessory pathway of the sympathetic nerve.^11^, ^12^ A lateral extension of electrocoagulation dissection along the upper and lower rib borders is recommended to block all potential pathways. However, the sympathetic ganglions are invisible in white light and there are many anatomical variations. Street *et al*.^13^ reported that 6.25% of ganglions were located below their associated intercostal spaces. Chung *et al*.^14^ reported that 78.8% T2 ganglion was located at the margin of the second intercostal space or upper margin of the third rib. Kim *et al*.^15^ revealed that 40.9% T3 ganglion was located at the upper border of the fourth rib or upon the fourth rib, and 81.8% T5 ganglion was located at the upper border of the fifth rib or upon the fifth rib. In this situation, when the sympathetic chain at R4 is dissected, we do not know whether the T4 sympathetic ganglion has been severed. To address this issue, a new applicable method for visualizing the sympathetic ganglion by means of NIR fluorescence imaging with ICG has been developed.^5^, ^16^

NIR fluorescence imaging‐guided surgery with ICG has been highlighted over the past few years. This technique has been used in many thoracic surgeries such as identification of the segmental border,^17^, ^18^ evaluation of gastric conduit perfusion,^19^ detection of bullous lesions,^20^, ^21^ detection of pulmonary nodules and solid tumor tissues,^22^ and localization of small pulmonary nodules.^23^ To the best of our knowledge, this is the first study to investigate the value of this technique during sympathectomy in the management of PPH.

ICG has proven to be a safe dye, but it can also cause adverse reactions. Yannuzzi *et al*.^7^ reported that there had been three (0.15%) mild adverse reactions, four (0.2%) moderate adverse reactions, and one (0.05%) severe adverse reaction in the 1923 ICG videoangiography tests. The dosage of ICG used in that study was 1–5 mg/kg. However, all patients received a 5 mg/kg of infusion of ICG 24 hours prior to surgery in our study. The dose of ICG that we used in our study was relatively high compared with the recommended dose for general use. According to the relevant literature, we found premedication with prednisolone and diphenhydramine could reduce the incidence of adverse reactions in sensitized patients before administration of iodine contrast medium.^24^ We used methylprednisolone (40 mg) as a premedication before ICG injection in our study for reasons of patient safety. The rate of mild adverse reaction was 0.70% in our study. One patient had a mild adverse reaction of subcutaneous extravasation after intravenous ICG injection, which caused mild stinging and a small area of skin which changed in color, but no tissue necrosis occurred. There were no moderate or severe adverse reactions caused by ICG injection. There was no significant difference in body temperature, heart rate and blood pressure before and after ICG injection. No significant difference was observed in the abnormal WBC count, ALT, AST, BUN and Cr levels before and after operation between the ICG group and Non‐ICG group. This study demonstrated that the intravenous injection of 5 mg/kg ICG is relatively safe. Similarly, many studies have also demonstrated that intravenous injection of 5 mg/kg ICG is feasible and safe.^6^, ^25^ Despite this, severe adverse reactions are possible^2^, ^4^, ^26^, ^27^ and therefore preparations for emergency rescue measures should be readily available.

In this study, the visibility rate of all patients was 96.7%. There was no significant difference in the visibility rate with regard to age, gender, height, weight, BMI, and the PPH grade. The visibility rate of sympathetic ganglions was influenced by multiple factors such as ICG dose, imaging timing, thickness of intercostal fat, sensitivity of the imaging system, and possibly the distance and angle between the target and NIR thoracoscope. In this study, we only discussed the safety and feasibility of intraoperative NIR imaging using ICG during sympathectomy, and did not discuss the anatomical variation rate of sympathetic ganglions and surgical results. The surgical outcomes are presented in another paper in which we found that downshift variation of sympathetic ganglia was associated with drier hands after sympathectomy. That paper was presented as an oral presentation in the 33th EACTS (European Association for Cardio‐Thoracic Surgery) Annual meeting (https://www.eacts.org/annual-meeting) and the 13th ISSS (International Society of Sympathetic Surgery) World Symposium (https://www.isssitaly2019.org).

Several limitations in the present study are acknowledged. First, the main limitation was the nonrandomized and retrospective nature of the study. Therefore, unknown confounding variables and selection biases, such as the selection of the operative method, may have biased the results. Second, we used methylprednisolone as a premedication before ICG injection in all ICG group patients for safety reasons which would have an effect on the incidence of adverse reactions. It would therefore make more sense to use it in those patients allergic to ICG. Third, using 142 patients for a safety trial is unnecessary. It is more important to show efficacy with such large numbers.

Despite the small sample size, we preliminarily conclude that applications of ICG based NIR fluorescence imaging for identifying the sympathetic ganglions is relatively safe and feasible. This technology may take the place of the rib‐oriented method as standard practice for the precise localization of sympathetic ganglions, and may improve the effect of sympathectomies.

## Disclosure

The authors declare no conflicts of interest.
